# Reproducibility of dietary intakes of macronutrients, specific food groups, and dietary patterns in 211 050 adults in the UK Biobank study

**DOI:** 10.1017/jns.2019.31

**Published:** 2019-10-29

**Authors:** Jennifer L. Carter, Sarah Lewington, Carmen Piernas, Kathryn Bradbury, Timothy J. Key, Susan A. Jebb, Matthew Arnold, Derrick Bennett, Robert Clarke

**Affiliations:** 1 Clinical Trial Service Unit and Epidemiological Studies Unit, Nuffield Department of Population Health, University of Oxford, Oxford, UK; 2 Medical Research Council Population Health Research Unit, Nuffield Department of Population Health, University of Oxford, Oxford, UK; 3 Nuffield Department of Primary Care Health Sciences, University of Oxford, Oxford, UK; 4 National Institute for Health Innovation, School of Population Health, The University of Auckland, Auckland, New Zealand; 5 Cancer Epidemiology Unit, Nuffield Department of Population Health, University of Oxford, Oxford, UK; 6 Department of Public Health and Primary Care, University of Cambridge, Cambridge, Cambridgeshire, UK

**Keywords:** 24-h recall, Dietary assessment, Dietary patterns, Food groups, Macronutrients, Reproducibility, ICC, intra-class correlation coefficient

## Abstract

To detect modest associations of dietary intake with disease risk, observational studies need to be large and control for moderate measurement errors. The reproducibility of dietary intakes of macronutrients, food groups and dietary patterns (vegetarian and Mediterranean) was assessed in adults in the UK Biobank study on up to five occasions using a web-based 24-h dietary assessment (*n* 211 050), and using short FFQ recorded at baseline (*n* 502 655) and after 4 years (*n* 20 346). When the means of two 24-h assessments were used, the intra-class correlation coefficients (ICC) for macronutrients varied from 0·63 for alcohol to 0·36 for polyunsaturated fat. The ICC for food groups also varied from 0·68 for fruit to 0·18 for fish. The ICC for the FFQ varied from 0·66 for meat and fruit to 0·48 for bread and cereals. The reproducibility was higher for vegetarian status (κ > 0·80) than for the Mediterranean dietary pattern (ICC = 0·45). Overall, the reproducibility of pairs of 24-h dietary assessments and single FFQ used in the UK Biobank were comparable with results of previous prospective studies using conventional methods. Analyses of diet–disease relationships need to correct for both measurement error and within-person variability in dietary intake in order to reliably assess any such associations with disease in the UK Biobank.

Diet has been estimated to account for about one-third of all deaths from CVD and cancer^(1,2)^. Recent research has continued to highlight the ongoing limitations in the methods used to reliably assess dietary intake in population studies using either 24-h dietary recalls or FFQ^(3,4)^. Epidemiological studies need to be large enough to detect modest differences in risk, and failure to appreciate the importance of within-person variability in dietary intake will result in underestimation of associations with disease in population studies. It is important to obtain repeat measurements in individuals of dietary intake at intervals of several months or years to control for random measurement error, within-person variability and true changes in diet, as all of these sources of variability contribute to misclassification of an individual's ‘usual’ dietary intake over a long period of time^(5)^. By controlling for these sources of variability, reliable estimates of the true associations between the ‘usual’ levels of dietary intake over a particular period and incident disease at the same or later time can be obtained^(5,6)^. Recent advances in technology now permit serial assessments of self-completed dietary questionnaires using web-based platforms in large-scale cohort studies. The potential for these digital technologies to improve the estimation of dietary intake, however, is ongoing and requires quantification prior to their use in population studies of diet and incident disease.

The UK Biobank is a prospective cohort study of 0·5 million adults and is one of the largest prospective studies with repeat 24-h assessments of dietary intake and FFQ. Measuring the reproducibility of these measurements, or the degree of consistency between the repeated 24-h assessments, will quantify the magnitude of within-person variability (i.e. change or variation in an individual's dietary intake and chance fluctuations in a person's recorded dietary intake that average to ‘true’ intake over repeat administrations)^(5,7,8)^. While reproducibility does not indicate how valid these 24-h assessments are (i.e. how accurately the measurements reflect true dietary intake), the estimates of reproducibility can still be used to correct for the underestimation of relative risks arising from random measurement errors in exposures (i.e. ‘regression dilution bias’) in order to calculate more reliable associations between ‘usual’ dietary intake and incident disease outcomes in the UK Biobank^(5,6)^. The aims of the present report were to estimate the reproducibility of dietary intakes of macronutrients, food groups and selected dietary patterns using serial web-based 24-h dietary assessments in 211 050 participants from the UK Biobank study who completed such assessments; and to compare measurements of intakes from food groups from the mean of 24-h dietary assessments with those estimated using a single short FFQ on all 0·5 million participants.

## Methods

**Fig. 1.**
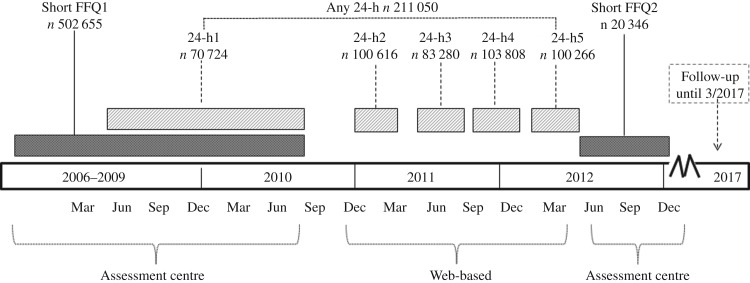
Timeline of UK Biobank dietary assessment measurement 2006–2015, including the 24-h dietary assessment (24-h) and the short FFQ.

The UK Biobank is a population-based prospective cohort study of 502 655 participants aged 40–69 years at recruitment between 2006 and 2010. Participants living within 25 miles (40·2 km) of twenty-two assessment centres across England, Wales and Scotland were identified through National Health Service (NHS) central registers (containing 98 % of the UK population). All eligible adults (about 9·2 million) were sent an invitation letter, with a response rate of about 5·5 %. Details of the study protocol have been published elsewhere^(9,10)^. UK Biobank participants completed two types of dietary assessments on several occasions between 2006 and 2013: a short FFQ and a 24-h dietary assessment (see Fig. 1). At baseline assessment, all participants completed a short FFQ (described below). Towards the end of the recruitment period an enhancement was added to the baseline assessment protocol so that, between April 2009 and September 2010, 70 724 participants attending their first assessment centre visit at recruitment completed a modified 24-h dietary assessment capturing the foods and beverages consumed during the previous 24 h (detailed below; see Fig. 1). Between February 2011 and April 2012, identical web-based 24-h dietary assessments were emailed on four separate occasions (cycles) over a 16-month period to 331 013 participants who provided a valid email address. Invitations were scheduled to maximise variation by season and day of the week. The response rate for the web-based assessment was 53 % (*n* 176 012), with a total of 211 050 participants completing at least one 24-h dietary assessment online or at the assessment centre. Of these, 84 175 (40 %) completed a single assessment; 48 129 (23 %) completed two assessments; 42 492 (20 %) completed three assessments; 30 488 (15 %) completed four assessments and 5766 (3 %) completed five assessments. The response rate varied between 26 and 33 % in each cycle, with some participants missing one cycle and then returning to complete a later cycle.

### Dietary measures from the 24-h dietary assessment

Unlike standard 24-h dietary recalls that ask respondents to report everything they ate in the previous day, the 24-h dietary assessments in the UK Biobank presented participants with a list of commonly consumed foods on which they indicated their consumption in the previous 24 h^(11–13)^. Consumption of up to 206 widely consumed foods and thirty-two types of drinks were recorded using the 24-h dietary assessment, which took approximately 15 min to complete (median 15 (interquartile range 11–19) min). Standard categories and descriptions were used for portion sizes of each food (for example, slices of bread), and participants were asked to enter the ingredients individually for composite dishes (for example, so spaghetti bolognaise would be recorded as separate items for pasta, meat and tomato-based sauce)^(12)^. An open text box was included at the end for additional items that were not included on the food list. Participants also indicated if their food consumption in the previous day was ‘typical’ or if they followed any special diets (for example, vegetarian or gluten-free). Total energy intake (kJ) and macronutrient (g) values were estimated for protein, total fat, saturated fat, polyunsaturated fat, carbohydrates, total sugars, starch, fibre and alcohol using previously validated methods^(12)^. Nutrient intakes were calculated using associated portion sizes for each food/beverage item and by multiplying the amount consumed by the nutrient composition, using standard food consumption tables for the UK^(14–23)^. Reproducibility was also calculated for six commonly consumed food groups: (i) meat (beef, lamb, pork, bacon, ham, sausage, liver, or chicken), (ii) cheese, (iii) bread and breakfast cereals, (iv) fruit (fresh or dried), (v) vegetables (excluding potatoes) and (vi) fish (oily and not oily).

### Dietary measures from the short FFQ

All 0·5 million UK Biobank participants completed a short computer touchscreen questionnaire at their initial assessment centre visit that included twenty-nine questions about their average diet over the previous 12 months, most of which asked about the frequency of consumption of the six food groups specified above. In addition, a sample of 20 346 participants completed a repeat assessment about 4 years later (Fig. 1). Typically, responses to the short FFQ included: never, less than once per week, once per week, 2–4 times per week, 5–6 times per week, or once or more daily. For fruit and vegetables, participants were asked to directly enter the average number of servings consumed each day; for bread and breakfast cereals, participants entered the average number of servings per week. Daily intake (g) for the short FFQ was calculated by multiplying the frequency of intake by standard portion size (g) in the UK (for example, eating chicken 5–6 times per week would have been (5·5/7) multiplied by 120 g)^(24)^. See Supplementary Methods for additional details.

Vegetarian and Mediterranean dietary patterns were also assessed as these dietary patterns have been more strongly associated with incident disease than individual nutrients. Participants were classified as consuming a vegetarian diet if they self-reported a vegetarian diet (24-h dietary assessment) or if they reported never eating any meat or fish (short FFQ). A Mediterranean-style diet score, as defined by previous research, was estimated for each cycle of the 24-h dietary assessment by summing the indicators for each of the following (diet score range 0–9): if participants consumed greater than the median servings per d for vegetables, legumes, fruit, nuts, fish, wholegrains and the ratio of dietary monounsaturated to saturated fat intake; less than the median intake for red and processed meat; and between 5 and 25 g per d for ethanol^(25,26)^.

### Statistical methods

The mean intakes of individual macronutrients, food groups and dietary patterns were calculated across 24-h dietary assessments. The Mediterranean diet score and macronutrient intakes were normally distributed, except for alcohol, which was transformed with a rank-based inverse normal transformation; distributions of food group intakes were similarly transformed. Reproducibility was assessed using intra-class correlation coefficients (ICC; i.e. the proportion of between-subject variance to the total variance) from a one-way ANOVA for all dietary intakes, except for a vegetarian dietary pattern, which was assessed with a weighted κ statistic because it was a dichotomous variable^(27)^. Since variation in daily dietary intake is expected to be high, the average of an increasing number of 24-h dietary assessments should reduce random error and provide a better estimate of the true ‘usual’ intake^(7,28)^. In the main analyses, the mean of the first two cycles of the 24-h dietary assessment was compared with the mean of the subsequent two cycles of the 24-h dietary assessment for participants who completed at least four 24-h assessments (*n* 36 254, 17·2 % of the total sample). To assess the impact of bias due to systematically under- or over-reporting energy intake, the ICC were estimated before and after adjustment for total energy intake using the nutrient residuals method (where residuals from a regression of energy on the nutrient of interest are used when calculating reproducibility in order to determine the variation in nutrient intake due only to the composition of the diet)^(29)^. Participants were excluded from analyses if they reported implausible values of total energy intake that were approximately >99 % of the sex-specific distribution (>20 000 kJ for men and >18 000 kJ for women; approximately 700 participants from each cycle). A minimum threshold of energy intake was not imposed since participants could have had a special dietary restriction prior to their 24-h dietary assessment.

Reproducibility was calculated using the 24-h dietary assessment separately for subgroups classified by season, age group, socio-economic group, BMI and if collected on weekdays or weekends. Ignoring the first cycle of the 24-h dietary assessment, which was administered over a 17-month period in the assessment centre, seasonality was analysed by comparing measurements from the online cycles 2–5 as these were each recorded over 3-month periods corresponding to different seasons. Cycle 2 was recorded in winter (February–April 2011), cycle 3 in summer (June–August 2011), cycle 4 in autumn (October–December 2011), and cycle 5 in spring (April–June 2012). Age at the first 24-h dietary assessment was classified into 5-year age bands (<45, 45–49, 50–54, 55–59, 60–64, 65+ years); and socio-economic group (assessed by the Townsend index of deprivation based on the participant's postcode at recruitment) was classified into quintiles^(30)^. BMI at recruitment was classified according to WHO standard categories (<18·5 kg/m^2^, underweight; 18·5–24·9 kg/m^2^, normal weight; 25–29·9 kg/m^2^, overweight; 30–34·9 kg/m^2^, obese class I; 35 kg/m^2^ +, obese class II/III). Analyses of day of the week compared assessments recorded concordantly on weekdays (Monday–Friday) or weekends (Saturday/Sunday), with those recorded discordantly on both. All analyses were stratified by sex, and overall estimates were calculated as an inverse-variance weighted average of the sex-specific estimates.

In addition to comparing estimates of reproducibility for food group intake measured on the 24-h assessment and short FFQ, correlation coefficients between the FFQ and 24-h dietary assessment were estimated to compare the rankings of usual food group consumption. This was done using a method that deattenuated the coefficients for random error in the 24-h dietary assessments (by accounting for the ICC of the variable number of 24-h assessments) in the subset of those completing at least two 24-h dietary assessments^(7,31)^. All analyses were conducted using Stata version 13^(32)^.

### Ethics

Research in the UK Biobank study was conducted according to the Declaration of Helsinki, and was approved by the North West Multi-Centre Research Ethics Committee (reference number 06/MRE08/65). During the touchscreen assessment at baseline, all participants provided informed consent to participate in the UK Biobank and be followed up, using a signature capture device.

## Results

### Population characteristics and intake of macronutrients

Selected baseline characteristics of the study participants completing at least one 24-h dietary assessment and those in the overall UK Biobank study population are shown in Table 1. Similar to the overall cohort, participants who completed a 24-h dietary assessment had a mean age of 56 (sd 8) years, 55 % were women, 96 % reported having White British ancestry and 21·3 % were in the most affluent quintile of the Townsend index. BMI and blood pressure were also similar in both groups, but the participants who completed the 24-h assessment were more likely to have a university degree (43 *v.* 33 %) and less likely to be current smokers (8 *v.* 11 %) compared with those in the overall cohort.

**Table 1.** Selected characteristics of all UK Biobank participants and the subset who completed at least one 24-h dietary assessment* (Numbers of participants and percentages; mean values and standard deviations)

	Overall (2006–2010)		24-h Assessment (2011–2012)	
	*n*	%	*n*	%
Participants (*n*)	502 655	–	211 050	–
Demographics				
Women	273 472	54	116 263	55
Men	229 183	46	94 787	45
Age (years)				
Mean	57		56	
sd	8		8	
White	472 837	95	201 319	96
Higher degree	161 215	33	89 779	43
Townsend index (% highest quintile)	100 691	20	44 940	21
Medical history				
Prior CVD	149 355	30	55 772	26
Prior diabetes	26 408	5	8869	4
Prior cancer	38 623	8	15 973	8
Lifestyle factors				
Alcohol intake				
Daily	101 792	20	48 155	23
1–4/week	244 791	49	105 092	50
<1/week	154 569	31	57 626	27
Days of moderate/vigorous exercise per week				
Mean	2·6		2·6	
sd	1·9		1·8	
Current smoking	52 990	11	16 555	8
Clinical measurements				
BMI (kg/m^2^)				
Mean	27		27	
sd	4·8		4·7	
Systolic blood pressure (mmHg)				
Mean	138		137	
sd	19		18	

* Number missing: Townsend index (overall *n* 627, 0·1 %; 24-h *n* 265, 0·1 %), exercise (overall *n* 12 227, 2·4 %; 24-h *n* 2083, 0·9 %), BMI (overall *n* 3106, 0·6 %; 24-h *n* 597, 0·3 %), systolic blood pressure (overall *n* 30135, 6·0 %; 24-h *n* 7845, 3·7 %).

The five cycles of the 24-h dietary assessment were recorded at intervals over a 28-month period (mean interval 5·9 (sd 2·0) months), and the absolute mean intake of macronutrients was similar across different cycles for both sexes (Supplementary Table S1). Although men consumed higher absolute amounts of energy and of each macronutrient than women, both sexes consumed similar proportions (% from energy) of macronutrients (Table 2).

**Table 2.** Dietary intake of macronutrients assessed by 24-h dietary assessment in the UK Biobank (Mean values and standard deviations)

	Overall (*n* 210 143)*				Men (*n* 94 311)				Women (*n* 115 832)			
	% Energy		g/d		% Energy		g/d		% Energy		g/d	
	Mean	sd	Mean	sd	Mean	sd	Mean	sd	Mean	sd	Mean	sd
Energy (kJ)	–		8733	2452	–		9464	2570	–		8136	2177
Total fat	32	7	77·1	29·2	32	7	82·8	31·0	32	7	72·4	26·8
Saturated fat	12	4	29·5	12·4	12	4	31·9	13·3	12	4	27·6	11·3
Polyunsaturated fat	6	2	14·2	7·1	6	2	15·2	7·5	6	2	13·5	6·7
Monounsaturated fat	14	3	33·3	13·0	14	3	35·7	13·8	14	3	31·3	12·0
Protein	16	4	81·6	24·7	16	4	86·4	26·2	17	4	77·8	22·5
Carbohydrates	46	8	251·3	80·4	46	8	269·2	84·3	47	8	236·7	73·9
Total sugars	22	7	119·3	47·8	21	7	124·5	50·1	23	7	115·1	45·4
Starch	22	6	121·8	46·1	23	6	134·6	48·7	22	6	111·4	41·1
Fibre density†	1·9	0·7	16·3	6·5	1·8	0·7	16·6	6·8	2·0	0·7	16·1	6·3
Alcohol	5	7	16·1	21·1	6	7	21·4	21·2	4	6	11·8	15·9

* For any participant with >1 measurement, the average over all 24-h cycles has been used.

† Fibre % energy is calculated as fibre density (g/MJ).

### Reproducibility of macronutrient intake

The reproducibility of single 24-h dietary assessment was modest, with ICC ranging from 0·23 for polyunsaturated fat to 0·46 for sugars (Table 3). As expected, the estimates of reproducibility were higher when the means of two cycles of 24-h assessments were compared; for most macronutrients reproducibility estimates were moderate and varied between 0·50 and 0·60 (range 0·36 for polyunsaturated fat to 0·63 for alcohol).

**Table 3.** Intra-class correlations of dietary intake of macronutrients estimated using single 24-h assessments and the average of two 24-h assessments, by sex*

	Single measurements			Cycles (1 + 2) *v.* (3 + 4)		
Nutrient (g)	Overall	Men	Women	Overall	Men	Women
*n*	210 143	94 311	115 832	36 140	15 852	20 288
Energy (kJ)	0·35	0·36	0·34	0·50	0·51	0·48
Total fat	0·33	0·35	0·31	0·48	0·50	0·46
Saturated fat	0·35	0·38	0·33	0·51	0·54	0·49
Polyunsaturated fat	0·23	0·24	0·22	0·36	0·37	0·35
Monounsaturated fat	0·31	0·33	0·30	0·46	0·48	0·44
Protein	0·30	0·30	0·29	0·43	0·43	0·42
Carbohydrates	0·40	0·42	0·38	0·55	0·57	0·53
Total sugars	0·46	0·48	0·44	0·61	0·63	0·59
Starch	0·34	0·34	0·33	0·48	0·49	0·47
Fibre	0·44	0·45	0·44	0·60	0·62	0·59
Alcohol	0·44	0·45	0·44	0·63	0·65	0·62

* Cycle (1 + 2) *v.* (3 + 4) intra-class correlations are computed from means of cycles 1 and 2, correlated with means of cycles 3 and 4.

Reproducibility was typically slightly higher in men than in women, and in participants aged ≥65 years than in younger participants, but did not vary substantially by season, day of the week, age, or index of deprivation. The reproducibility was also unaltered by adjustment for total energy intake so the nutrient residuals method is reported in the Supplement, and the main results present the unadjusted estimates (Supplementary Tables S2–S4). The only exception was alcohol intake where reproducibility was greater if both measurements were recorded at weekends rather than on weekdays (for example, 0·60 *v.* 0·47 for men). The reproducibility for alcohol also increased with age (for example, from 0·38 in men <45 years to 0·52 in those aged ≥65 years). Participants who were underweight reported the highest reproducibility for macronutrient intake, with small differences seen across other groups categorised by BMI (Supplementary Table S5).

### Reproducibility of selected food groups and dietary patterns

The average dietary intake of food groups and dietary patterns from the short FFQ and 24-h assessments can be seen in Supplementary Table S6. Overall, the reproducibility of consumption of food groups assessed using a single 24-h dietary assessment was modest and varied between 0·10 (fish) to 0·52 (fruit) (Table 4). Reproducibility improved when comparing the means of two 24-h dietary assessments (range 0·18 for fish to 0·68 for fruit), but was still modest for several food groups like meat, cheese and fish. The reproducibility for food groups assessed using the short FFQ was generally higher, with most ICC around 0·60, although two food groups reported higher reproducibility on the 24-h assessments (bread/cereal and fruit). Comparisons of the intakes of food groups assessed using the short FFQ and the averages of all 24-h dietary assessments (if ≥2 cycles completed) demonstrated moderate agreement, with deattenuated correlation coefficients varying from 0·38 for vegetables to 0·63 for fruit.

**Table 4.** Intra-class correlations of food groups and dietary patterns in UK Biobank 24-h dietary assessment (*n* 211 050; ‘24-h’) and short FFQ (*n* 502 655; ‘FFQ’)*

	Overall				Men				Women			
	24-h		FFQ1		24-h		FFQ1		24-h		FFQ1	
	*v*. 24-h	(1 + 2) *v*. (3 + 4)	*v*. FFQ2	*v*. 24-h†	*v*. 24-h	(1 + 2) *v*. (3 + 4)	*v*. FFQ2	*v*. 24-h†	*v*. 24-h	(1 + 2) *v*. (3 + 4)	*v*. FFQ2	*v*. 24-h†
*n*	126 875	36 254	20 346	126 875	56 142	15 915	9940	56 142	70 733	20 339	10 406	70 733
Food groups												
Total meat	0·24	0·38	0·66	0·50	0·23	0·37	0·65	0·46	0·24	0·40	0·68	0·53
Cheese	0·22	0·38	0·63	0·51	0·24	0·39	0·64	0·53	0·21	0·36	0·61	0·49
Fish	0·10	0·18	0·65	0·47	0·10	0·18	0·64	0·50	0·09	0·18	0·66	0·45
Bread and cereals	0·37	0·55	0·48	0·52	0·37	0·55	0·49	0·51	0·37	0·54	0·47	0·54
Fruit	0·52	0·68	0·66	0·63	0·53	0·70	0·67	0·64	0·51	0·66	0·64	0·61
Vegetables	0·31	0·46	0·59	0·38	0·31	0·46	0·59	0·38	0·31	0·47	0·59	0·38
Dietary patterns												
Mediterranean‡	0·30	0·45			0·30	0·47			0·29	0·44		
Vegetarian§	0·81	0·84	0·92	0·69	0·81	0·84	0·93	0·69	0·81	0·84	0·91	0·68

* *n* is the maximum number of participants in each column, with each food group reporting <1 % missing.

† FFQ1 *v*. 24-h is the deattenuated correlation coefficient between baseline FFQ measurements and mean intake on 24-h assessments for those with 2+ cycles.

‡ Greater than median intake (servings/d) for vegetables, legumes, fruit, nuts, fish, wholegrains, ratio of monounsaturated to saturated fat; less than median intake for red and processed meat; and between 5 and 25 g/d for ethanol.

§ Reproducibility coefficients for the vegetarian dietary pattern are κ statistics.

Similar to estimates for food groups, the reproducibility of the Mediterranean dietary pattern was 0·45 when the means of two pairs of 24-h dietary assessments were compared. There was very good agreement when a vegetarian dietary pattern was reported using either the short FFQ or the 24-h dietary assessment (κ > 0·80).

As a sensitivity analysis, reproducibility was estimated after omitting participants reporting an ‘atypical’ diet in the previous 24 h, and the levels of reproducibility on 24-h dietary assessments improved by 0·03–0·04 for all types of dietary intake.

## Discussion

The reproducibility of the self-completed 24-h dietary assessments and the short FFQ was assessed in the large prospective UK Biobank study. The reproducibility of dietary intake assessed using the mean of two 24-h dietary assessments was moderate and generally varied between 0·50 and 0·60 for macronutrients. However, the reproducibility estimates of selected food groups using the mean of two 24-h dietary assessments were variable, and generally lower (albeit not consistently) than estimates reported using the short FFQ. The reproducibility of the vegetarian dietary pattern was high irrespective of the type of dietary assessment, although the reproducibility of the Mediterranean dietary pattern was lower and comparable with those for food groups and macronutrients.

As expected, the reproducibility estimates of all measures of dietary intake assessed solely using a pair of single 24-h dietary assessment in the UK Biobank were generally modest (between 0·30 and 0·40) due to measurement error and the day-to-day variation in dietary intake, consistent with estimates from previous studies^(28,33–39)^. While a single 24-h dietary assessment does not provide an adequate indication of usual long-term dietary intake for individuals, such measurements have still been used in some observational studies of diet and disease^(40)^, despite clear evidence from previous research that the means of several 24-h dietary assessments for individuals are required for reliable estimation of absolute values for usual intake of most macronutrients^(28,41–43)^. In the present study, the reproducibility of intake of macronutrients using the mean of two 24-h dietary assessments was higher, and approximately comparable with those reported by FFQ widely used in previous studies of diet and disease, with reproducibility estimates of around 0·50–0·60^(5)^. This suggests that averaging measurements from at least two 24-h assessments may be similar to FFQ for capturing the consistency of dietary intake over longer periods of time. The reproducibility coefficients for the mean of two 24-h dietary assessments were similar to those for blood pressure and total cholesterol, which are typically in the range 0·60–0·70^(5,6)^. Hence, the mean of at least two 24-h dietary assessments in the UK Biobank have a similar magnitude of random measurement error or within-person variability and will be informative for analyses of associations with disease in observational studies. However, while methods used to correct for within-person random error are well established, the sources of error in dietary intake are likely to be more complex than for biochemical measurements, and validity studies with recovery or concentration biomarkers of dietary intake may be necessary to fully correct for such errors^(8)^.

While the within-person variability in a single 24-h dietary assessment was substantial in the UK Biobank, it did not appear to vary by subgroups such as age, sex, season, day of the week or index of deprivation. However, previous studies addressing these potential sources of variation reported mixed findings^(34,43–47)^. Previous studies have also reported that reproducibility of daily intake of food groups on 24-h assessments was generally lower than for macronutrients^(5,34)^. Similar findings were observed in the present study for some of the food groups, although certain foods that were eaten regularly on a daily basis, like fruit, were more reproducible on the 24-h assessments than most macronutrients. Estimates of reproducibility for intake of most food groups were also slightly higher in the short FFQ than in the 24-h assessments in this study. However, only a small number of food groups (*n* 6) were measured in both types of assessments and could be compared, and it would be expected that, for example, fish would be better captured with a FFQ than two 24-h assessments. Overall, agreement between the two types of dietary assessment was moderate, and consistent with findings of a previous report that suggested the short FFQ adequately discriminated between high and low intakes on the 24-h dietary assessment in the UK Biobank for selected food groups^(48)^. The level of agreement reported between dietary assessments used in this study was also comparable with estimates reported between traditional 24-h recalls and FFQ reported in previous studies^(49–51)^, indicating that measurements of food group intake in the non-traditional UK Biobank dietary assessments are acceptable and suitable for use in prospective analyses. However, some of the level of agreement between the FFQ and 24-h assessment may be due to correlated errors, and since the dietary assessments in the UK Biobank have not yet been compared with objective measures of dietary intake such as recovery biomarkers, the validity of these assessments has not yet been established.

Reproducibility of a vegetarian dietary pattern was high if self-identified (24-h assessment) or if indicated by non-consumption of meat and fish in the short FFQ. While no other estimates of reproducibility of the Mediterranean diet could be found, previous research using FFQ to define multifaceted ‘prudent’ or ‘Western’ dietary patterns have reported reproducibility estimates around 0·70^(52)^. However, the estimate of reproducibility for the mean of two 24-h dietary assessments of the Mediterranean dietary pattern in this study was lower, and similar to the less reproducible macronutrients with an ICC of 0·45. Components of the Mediterranean diet, like nuts, fish or olive oil, may not be consumed frequently enough in the UK for a small number of 24-h dietary assessments to record reliably, which should be considered if researchers plan to assess associations of this dietary pattern with incident disease.

The findings in the present study indicating that the 24-h dietary assessments have acceptable reproducibility, provided at least two assessments are used, have important implications for analysis of UK Biobank dietary intake data. The UK Biobank is one of the largest studies with multiple 24-h dietary assessments, with over 78 000 participants completing at least three dietary assessments on different occasions. A recent analysis suggested that use of at least three 24-h dietary recalls had the best calibration with true intakes measured by recovery biomarkers when comparing serial measurements using FFQ and 24-h recalls^(4)^. However, the strength of the associations between usual dietary intake and disease risk in observational studies like the UK Biobank will still be underestimated if uncorrected for within-person variability in dietary assessments. Correction for such random within-person error (but not systematic error, which is likely to be still present in dietary assessments) can be done, as in the present report, using reproducibility measurements from a sample of the original cohort and does not require a validation study with a ‘gold standard’^(6)^. The large size of the UK Biobank substudy is an additional strength as it allows reliable estimates of these correction factors^(27)^.

One strength of the 24-h dietary assessments used in the UK Biobank was that measurements were recorded using a web-based platform in a short period of time, with automatic coding of nutrients. This web-based platform limited the burden to participants and researchers (and hence improved the acceptability), and therefore permitted assessments to be administered to a large number of participants^(13)^. However, the web-based format may discourage or prohibit certain types of participants from completing it, particularly participants with ill health or poor computer literacy^(53–55)^. Previous analyses of 24-h assessments in the UK Biobank reported that participants completing multiple assessments tended to be older and more educated, but that there were small differences by sex and deprivation^(13)^. Furthermore, without the assistance of trained interviewers, participants may have omitted some food items and required assistance for portion sizes^(28)^. Collectively, this may have increased the measurement error in the 24-h dietary assessments. However, studies indicated comparable results between a web-based 24-h dietary assessment method and an interviewer-administered 24-h dietary assessment completed on the same day^(12,55)^. The web-based 24-h dietary assessment also suffers from the standard measurement issues common to many dietary assessments, all of which may have had an impact on reproducibility, such as incomplete information on ingredients in composite dishes, discrepancies in how participants reported portion sizes, and general issues with memory recall^(28)^. Since 24-h dietary assessments in the UK Biobank differed from standard ‘24-h recalls’ that are commonly used in nutritional epidemiology, some foods may have been missed, as only 206 foods and 32 beverages were surveyed. However, total energy intake as computed from the 24-h assessments was not notably low, suggesting that few important foods were missed.

While the mean of two 24-h dietary assessments in the UK Biobank had acceptable reproducibility, this could still lead to underestimation of the strength of associations with disease by up to 50 % (as correction factors from the ICC are still around 0·50–0·60). Previous reports on the recommended methods to correct for measurement error by Willett^(7)^ and Bennett *et al*.^(8)^ include several approaches for minimising the effects of regression dilution bias that can arise from both measurement error and within-person variability assessed in the present study. Therefore, it is important that analyses of diet−disease relationships take account of both measurement error and within-person variability as diet may be an even more important determinant of chronic disease risk than has previously been realised.

## Supplementary material

For supplementary material accompanying this paper visit https://doi.org/10.1017/jns.2019.31. click here to view supplementary material
