# Supplementary material for: Reproducibility of dietary intakes of macronutrients, specific food groups, and dietary patterns in 211 050 adults in the UK Biobank study
Source: J Nutr Sci. 2019 Oct 29;8:e34. doi: 10.1017/jns.2019.31 (PMC6842574; doi:10.1017/jns.2019.31)
Supplement: Supplementary file 1 [file S2048679019000314sup001.doc]

**Supplementary material**

**Supplementary Methods.** Calculation of food groups for both 24-hour dietary assessments and short FFQ ^a^

| **Food Group**  **FFQ** | | **Portion Size (g) FFQ** | **Food Group**  **24-Hr Assessment** | **Portion Size (g)**  **24-Hr Assessment** |
| --- | --- | --- | --- | --- |
| *Total Meat* | |  |  |  |
|  | Beef | 120 | Beef (e.g. roast, steak, mince, curry, burger) | 120 |
|  | Pork | 120 | Pork (e.g. roast, chops, sweet and sour) | 120 |
|  | Lamb | 120 | Lamb or mutton (e.g. roast, chops, stew, burger) | 120 |
|  | Poultry | 130 | Chicken or turkey (e.g. roast, drumsticks, curry) | 130 |
|  | Processed meat | 90 | Sausage | 30 |
|  |  |  | Bacon | 46 |
|  |  |  | Ham, Parma ham, salami, pastrami, cured meats | 23 |
|  | Other^b^ |  | Chicken or turkey in breadcrumbs or deep fried (e.g. nuggets, KFC) | 100 |
|  |  |  | Liver or liver pate | 70 |
|  |  |  | Other meat (e.g. duck, goose, kidney) | 100 |
| *Fish* | |  |  |  |
|  | Oily fish | 100 | Oily fish (e.g. salmon, tinned salmon, herring, mackerel, sardines, fresh tuna steak) | 100 |
|  | Non-oily fish | 134 | Tinned tuna | 92 |
|  |  |  | Breaded fish (e.g. fish fingers) or fish cakes | 56 |
|  |  |  | Battered fish | 190 |
|  |  |  | White fish (e.g. cod, haddock, fish pie) | 120 |
| *Fruit* | |  |  |  |
|  | Fresh fruit | 100 | Mixed fruit | 105 |
|  |  |  | Apple | 100 |
|  |  |  | Banana | 100 |
|  |  |  | Berries | 40 |
|  |  |  | Cherries | 24 |
|  |  |  | Grapefruit | 160 |
|  |  |  | Grapes | 100 |
|  |  |  | Mango | 100 |
|  |  |  | Melon | 180 |
|  |  |  | Orange | 120 |
|  |  |  | Satsuma | 70 |
|  |  |  | Peach | 150 |
|  |  |  | Pear | 120 |
|  |  |  | Pineapple | 80 |
|  |  |  | Plum | 55 |
|  |  |  | Other fruit | 60 |
|  | Dried fruit | 8 | Prunes | 60 |
|  |  |  | Dried fruit | 60 |
|  |  |  | Dried fruit in breakfast cereal | 14 |
| *Cheese* | | 40 | Low fat hard cheese | 40 |
|  |  |  | Hard cheese | 40 |
|  |  |  | Soft cheese | 40 |
|  |  |  | Blue cheese | 35 |
|  |  |  | Low fat cheese spread | 15 |
|  |  |  | Cheese spread | 15 |
|  |  |  | Cottage cheese | 60 |
|  |  |  | Feta | 40 |
|  |  |  | Mozzarella | 40 |
|  |  |  | Goat cheese | 40 |
|  |  |  | Other cheese | 40 |
| *Vegetables* | |  |  |  |
|  | Raw | 30 | Salad | 66 |
|  | Cooked | 40 | Mixed vegetables | 90 |
|  |  |  | Baked beans | 135 |
|  |  |  | Veg pieces | 90 |
|  |  |  | Coleslaw | 120 |
|  |  |  | Pulses | 70 |
|  |  |  | Avocado | 136 |
|  |  |  | Broadbeans | 70 |
|  |  |  | Green beans | 70 |
|  |  |  | Beetroot | 48 |
|  |  |  | Broccoli | 80 |
|  |  |  | Squash | 130 |
|  |  |  | Cabbage | 90 |
|  |  |  | Carrots | 60 |
|  |  |  | Cauliflower | 90 |
|  |  |  | Celery | 30 |
|  |  |  | Courgette | 90 |
|  |  |  | Cucumber | 60 |
|  |  |  | Garlic | 5 |
|  |  |  | Leeks | 80 |
|  |  |  | Lettuce | 35 |
|  |  |  | Mushrooms | 44 |
|  |  |  | Onion | 60 |
|  |  |  | Parsnip | 65 |
|  |  |  | Peas | 65 |
|  |  |  | Peppers | 160 |
|  |  |  | Spinach | 90 |
|  |  |  | Sprouts | 90 |
|  |  |  | Corn | 43 |
|  |  |  | Sweet potato | 130 |
|  |  |  | Tomato fresh | 85 |
|  |  |  | Tomato tinned | 135 |
|  |  |  | Turnip | 60 |
|  |  |  | Watercress | 20 |
|  |  |  | Other vegetables | 60 |
| *Bread & Breakfast Cereals* | | | |  |
|  | Bread | 36 | White | 36 |
|  |  |  | Mixed | 36 |
|  |  |  | Wholemeal | 36 |
|  |  |  | Other | 36 |
|  | Cereal^c^ | 203 | Porridge | 203 |
|  |  | 100 | Muesli | 100 |
|  |  |  | Oat crunch | 100 |
|  |  | 38 | Sweet | 38 |
|  |  |  | Sweet with dried fruit | 52 |
|  |  |  | Plain | 30 |
|  |  |  | Plain with dried fruit | 44 |
|  |  | 50 | Bran | 50 |
|  |  |  | Bran with dried fruit | 52 |
|  |  | 44 | Wholewheat | 44 |
|  |  |  | Other | 44 |
|  |  |  | Other with dried fruit | 55 |

^a^ Some serving sizes differed on the questions included on the FFQ and 24-hr assessments so the portion sizes (g) will reflect this.  ^b^ Not included in FFQ. ^c^ Types of cereal in FFQ are matched with those on the 24-hr assessment as applicable.

Daily intake (g) of each food group on the short food frequency questionnaire (FFQ) was calculated by multiplying the frequency of intake per day by standard portion sizes (g) in the UK (for example, eating chicken 5-6 times a week would have been [5.5/7] multiplied by 120 grams). Daily intake (g) of each individual food on the 24-hr dietary assessment was calculated by multiplying the number of servings consumed in the previous 24 hours by the standard portion size (g) in the UK. Daily intakes of individual foods in the 24-hr dietary assessment were then summed together to reflect the broader food group category indicated on the FFQ.

**Supplementary Table S1.** Dietary intake of macronutrients from the 24-hour dietary assessment in UK Biobank

|  | Mean (SD) for individuals | | | | | | | | | | | | |
| --- | --- | --- | --- | --- | --- | --- | --- | --- | --- | --- | --- | --- | --- |
|  | Overall^b^ | | Cycle 1: Apr.09-Sep.10 | | Cycle 2: Feb.11-Apr.11 | | | Cycle 3: Jun.11-Sep.11 | | Cycle 4: Oct.11-Dec.11 | | Cycle 5: Apr.12-Jun.12 | |
| Nutrient | Grams | % Energy | Grams | % Energy | Grams | | % Energy | Grams | % Energy | Grams | % Energy | Grams | % Energy |
| N |  |  |  |  |  | |  |  |  |  |  |  |  |
| Women | 115,832 |  | 38,780 |  | 55,265 | |  | 46,074 |  | 57,179 |  | 55,765 |  |
| Men | 94,311 |  | 31,298 |  | 44,641 | |  | 36,573 |  | 45,958 |  | 43,731 |  |
| Energy (kJ) |  |  |  |  |  | |  |  |  |  |  |  |  |
| Women | 8,136 (2,177) | | 8,040 (2,539) | | 8,200 (2,455) | | | 8,180 (2,458) | | 8,096 (2,428) | | 8,203 (2,463) | |
| Men | 9,464 (2,570) | | 9,480 (3,013) | | 9,469 (2,836) | | | 9,478 (2,880) | | 9,429 (2,841) | | 9,485 (2,869) | |
| Protein |  |  |  |  |  |  | |  |  |  |  |  |  |
| Women | 77.8 (22.5) | 0.17 (0.04) | 76.5 (26.4) | 0.17 (0.05) | 78.4 (25.7) | | 0.17 (0.04) | 78.1 (26.2) | 0.17 (0.05) | 77.5 (25.3) | 0.17 (0.04) | 78.1 (25.9) | 0.17 (0.04) |
| Men | 86.4 (26.2) | 0.16 (0.04) | 86.0 (31.1) | 0.16 (0.04) | 86.7 (29.2) | | 0.16 (0.04) | 86.2 (30.0) | 0.16 (0.04) | 86.2 (29.2) | 0.16 (0.04) | 86.5 (29.6) | 0.16 (0.04) |
| Total Fat |  |  |  |  |  | |  |  |  |  |  |  |  |
| Women | 72.4 (26.8) | 0.32 (0.07) | 70.5 (31.2) | 0.32 (0.08) | 72.9 (30.8) | | 0.32 (0.08) | 72.9 (30.9) | 0.32 (0.08) | 72.3 (30.3) | 0.33 (0.08) | 74.1 (31.0) | 0.33 (0.08) |
| Men | 82.8 (31.0) | 0.32 (0.07) | 81.9 (36.1) | 0.31 (0.08) | 83.2 (34.7) | | 0.32 (0.08) | 82.8 (35.3) | 0.32 (0.08) | 83.1 (34.8) | 0.32 (0.08) | 84.0 (35.4) | 0.32 (0.08) |
| Saturated Fat |  |  |  |  |  | |  |  |  |  |  |  |  |
| Women | 27.6 (11.3) | 0.12 (0.04) | 26.8 (13.1) | 0.12 (0.04) | 27.7 (12.9) | | 0.12 (0.04) | 27.7 (13.1) | 0.12 (0.04) | 27.8 (12.9) | 0.13 (0.04) | 28.2 (13.1) | 0.13 (0.04) |
| Men | 31.9 (13.3) | 0.12 (0.04) | 31.5 (15.4) | 0.12 (0.04) | 31.9 (14.8) | | 0.12 (0.04) | 31.8 (15.1) | 0.12 (0.04) | 32.1 (14.8) | 0.12 (0.04) | 32.3 (15.1) | 0.12 (0.04) |
| Polyunsaturated Fat |  |  |  |  |  | |  |  |  |  |  |  |  |
| Women | 13.5 (6.7) | 0.06 (0.02) | 13.3 (8.1) | 0.06 (0.03) | 13.6 (7.9) | | 0.06 (0.03) | 13.5 (7.9) | 0.06 (0.03) | 13.4 (7.9) | 0.06 (0.03) | 13.8 (8.0) | 0.06 (0.03) |
| Men | 15.2 (7.5) | 0.06 (0.02) | 15.0 (9.0) | 0.06 (0.03) | 15.3 (8.8) | | 0.06 (0.03) | 15.2 (8.7) | 0.06 (0.03) | 15.2 (8.8) | 0.06 (0.03) | 15.4 (8.9) | 0.06 (0.03) |
| Carbohydrates |  |  |  |  |  | |  |  |  |  |  |  |  |
| Women | 236.7 (73.9) | 0.47 (0.08) | 237.8 (86.2) | 0.48 (0.09) | 238.4 (82.3) | | 0.47 (0.09) | 236.2 (82.8) | 0.46 (0.09) | 234.5 (81.9) | 0.47 (0.09) | 236.4 (82.6) | 0.46 (0.09) |
| Men | 269.2 (84.3) | 0.46 (0.08) | 272.3 (98.1) | 0.46 (0.09) | 268.9 (92.1) | | 0.46 (0.09) | 268.1 (92.4) | 0.46 (0.09) | 267.1 (92.2) | 0.46 (0.09) | 267.8 (92.2) | 0.46 (0.09) |
| Total Sugars |  |  |  |  |  | |  |  |  |  |  |  |  |
| Women | 115.1 (45.4) | 0.23 (0.07) | 116.3 (52.9) | 0.23 (0.08) | 115.9 (50.0) | | 0.23 (0.08) | 115.0 (49.6) | 0.23 (0.08) | 112.8 (49.4) | 0.22 (0.08) | 114.4 (49.3) | 0.23 (0.08) |
| Men | 124.5 (50.1) | 0.21 (0.07) | 126.6 (57.7) | 0.22 (0.08) | 124.1 (54.1) | | 0.21 (0.07) | 124.5 (54.3) | 0.21 (0.08) | 122.3 (53.4) | 0.21 (0.07) | 123.3 (53.5) | 0.21(0.07) |
| Starch |  |  |  |  |  | |  |  |  |  |  |  |  |
| Women | 111.4 (41.1) | 0.22 (0.06) | 111.4 (48.2) | 0.22 (0.07) | 112.3 (46.6) | | 0.22 (0.07) | 110.0 (46.5) | 0.22 (0.07) | 111.9 (46.3) | 0.22 (0.07) | 111.2 (46.6) | 0.22 (0.07) |
| Men | 134.6 (48.7) | 0.23 (0.06) | 135.9 (58.1) | 0.23 (0.07) | 135.0 (54.2) | | 0.23 (0.07) | 132.8 (54.2) | 0.23 (0.07) | 135.1 (54.3) | 0.23 (0.07) | 134.3 (54.4) | 0.23 (0.07) |
| Fiber ^a^ |  |  |  |  |  | |  |  |  |  |  |  |  |
| Women | 16.1 (6.3) | 2.04 (0.74) | 16.3 (7.4) | 2.09 (0.88) | 16.3 (7.0) | | 2.05 (0.82) | 16.1 (7.0) | 2.02 (0.83) | 16.1 (6.9) | 2.04 (0.81) | 16.0 (6.8) | 2.01 (0.80) |
| Men | 16.6 (6.8) | 1.80 (0.67) | 16.7 (7.9) | 1.81 (0.78) | 16.7 (7.4) | | 1.81 (0.74) | 16.6 (7.5) | 1.79 (0.75) | 16.7 (7.4) | 1.81 (0.73) | 16.5 (7.3) | 1.79 (0.73) |
| Alcohol |  |  |  |  |  | |  |  |  |  |  |  |  |
| Women | 11.8 (15.9) | 0.04 (0.06) | 11.0 (17.6) | 0.04 (0.06) | 12.0 (18.0) | | 0.04 (0.06) | 12.8 (18.4) | 0.05 (0.06) | 11.8 (17.6) | 0.04 (0.06) | 11.9 (17.8) | 0.04 (0.06) |
| Men | 21.4 (21.2) | 0.06 (0.07) | 21.7 (28.9) | 0.07 (0.09) | 21.0 (27.3) | | 0.06 (0.08) | 22.6 (28.5) | 0.07 (0.08) | 21.0 (27.4) | 0.06 (0.08) | 21.3 (27.5) | 0.06 (0.08) |

^a^Fibre % Energy is calculated as fibre density (g/MJ). **^b^** For any participant with >1 measurement, the average over all measurement has been used.

**Supplementary Table S2.** Intra-class correlations (ICC) between repeat measurements of macronutrients in UK Biobank’s 24-hour dietary assessment, unadjusted and adjusted for total energy intake ^a^.

| Nutrient (g) | Unadjusted  ICC | Adjusted  ICC | Unadjusted  Weekday | Adjusted  Weekday | Unadjusted Weekend | Adjusted  Weekend | Unadjusted  Weekday vs Weekend | Adjusted  Weekday vs Weekend |
| --- | --- | --- | --- | --- | --- | --- | --- | --- |
| N Women | 70,733 | 70,733 | 33,402 | 33,402 | 1,555 | 1,555 | 35,776 | 35,776 |
| Men | 56,142 | 56,142 | 27,408 | 27,408 | 1,200 | 1,200 | 27,534 | 27,534 |
| Energy (kJ) |  |  |  |  |  |  |  |  |
| Women | 0.34 |  | 0.35 |  | 0.37 |  | 0.32 |  |
| Men | 0.36 |  | 0.37 |  | 0.36 |  | 0.35 |  |
| Protein |  |  |  |  |  |  |  |  |
| Women | 0.29 | 0.26 | 0.30 | 0.27 | 0.35 | 0.27 | 0.27 | 0.25 |
| Men | 0.30 | 0.26 | 0.31 | 0.26 | 0.30 | 0.28 | 0.28 | 0.24 |
| Total Fat |  |  |  |  |  |  |  |  |
| Women | 0.31 | 0.29 | 0.32 | 0.29 | 0.31 | 0.28 | 0.30 | 0.29 |
| Men | 0.35 | 0.32 | 0.35 | 0.33 | 0.31 | 0.32 | 0.34 | 0.32 |
| Saturated Fat |  |  |  |  |  |  |  |  |
| Women | 0.33 | 0.34 | 0.34 | 0.34 | 0.30 | 0.31 | 0.32 | 0.33 |
| Men | 0.38 | 0.37 | 0.38 | 0.38 | 0.34 | 0.37 | 0.37 | 0.36 |
| Polyunsaturated Fat |  |  |  |  |  |  |  |  |
| Women | 0.22 | 0.18 | 0.22 | 0.18 | 0.24 | 0.17 | 0.21 | 0.18 |
| Men | 0.24 | 0.20 | 0.24 | 0.20 | 0.21 | 0.20 | 0.23 | 0.20 |
| Monounsaturated Fat |  |  |  |  |  |  |  |  |
| Women | 0.30 | 0.24 | 0.30 | 0.24 | 0.31 | 0.24 | 0.29 | 0.24 |
| Men | 0.33 | 0.27 | 0.34 | 0.27 | 0.30 | 0.25 | 0.32 | 0.26 |
| Carbohydrates |  |  |  |  |  |  |  |  |
| Women | 0.38 | 0.35 | 0.39 | 0.35 | 0.38 | 0.32 | 0.37 | 0.35 |
| Men | 0.42 | 0.41 | 0.43 | 0.41 | 0.40 | 0.42 | 0.40 | 0.41 |
| Total Sugars |  |  |  |  |  |  |  |  |
| Women | 0.44 | 0.42 | 0.45 | 0.42 | 0.43 | 0.41 | 0.42 | 0.40 |
| Men | 0.48 | 0.45 | 0.49 | 0.46 | 0.46 | 0.43 | 0.46 | 0.44 |
| Starch |  |  |  |  |  |  |  |  |
| Women | 0.33 | 0.28 | 0.34 | 0.29 | 0.33 | 0.28 | 0.32 | 0.28 |
| Men | 0.34 | 0.31 | 0.35 | 0.32 | 0.33 | 0.30 | 0.32 | 0.29 |
| Fibre |  |  |  |  |  |  |  |  |
| Women | 0.44 | 0.43 | 0.45 | 0.44 | 0.49 | 0.45 | 0.42 | 0.42 |
| Men | 0.45 | 0.45 | 0.46 | 0.45 | 0.44 | 0.46 | 0.45 | 0.44 |
| Alcohol |  |  |  |  |  |  |  |  |
| Women | 0.45 | 0.47 | 0.45 | 0.47 | 0.58 | 0.60 | 0.43 | 0.45 |
| Men | 0.46 | 0.49 | 0.47 | 0.50 | 0.60 | 0.60 | 0.44 | 0.47 |

^a^ All ICCs are derived from those with 2 or more measurements.

**Supplementary Table S3.** Reproducibility of 24-hour dietary assessment cycles conducted in different seasons in UK Biobank. Intra-class correlations unadjusted for energy intake are presented ^a^

|  | Winter vs | | |  | Summer vs | |  | Spring vs |
| --- | --- | --- | --- | --- | --- | --- | --- | --- |
| Nutrient (g) | Spring | Summer | Autumn |  | Spring | Autumn |  | Autumn |
| N Women | 31,078 | 27,041 | 33,436 |  | 27,972 | 29,981 |  | 35,914 |
| Men | 24,287 | 21,465 | 26,722 |  | 21,879 | 23,759 |  | 28,246 |
| Energy (kJ) |  |  |  |  |  |  |  |  |
| Women | 0.33 | 0.34 | 0.36 |  | 0.36 | 0.34 |  | 0.35 |
| Men | 0.35 | 0.36 | 0.38 |  | 0.38 | 0.37 |  | 0.37 |
| Protein |  |  |  |  |  |  |  |  |
| Women | 0.29 | 0.30 | 0.30 |  | 0.31 | 0.29 |  | 0.31 |
| Men | 0.30 | 0.30 | 0.31 |  | 0.30 | 0.30 |  | 0.31 |
| Total Fat |  |  |  |  |  |  |  |  |
| Women | 0.31 | 0.32 | 0.33 |  | 0.33 | 0.31 |  | 0.32 |
| Men | 0.33 | 0.34 | 0.37 |  | 0.36 | 0.36 |  | 0.35 |
| Saturated Fat |  |  |  |  |  |  |  |  |
| Women | 0.32 | 0.34 | 0.35 |  | 0.34 | 0.34 |  | 0.34 |
| Men | 0.36 | 0.38 | 0.40 |  | 0.39 | 0.39 |  | 0.38 |
| Polyunsaturated Fat |  |  |  |  |  |  |  |  |
| Women | 0.22 | 0.22 | 0.23 |  | 0.24 | 0.22 |  | 0.22 |
| Men | 0.23 | 0.24 | 0.25 |  | 0.25 | 0.24 |  | 0.24 |
| Monounsaturated Fat |  |  |  |  |  |  |  |  |
| Women | 0.29 | 0.31 | 0.31 |  | 0.31 | 0.30 |  | 0.31 |
| Men | 0.32 | 0.33 | 0.35 |  | 0.34 | 0.34 |  | 0.34 |
| Carbohydrates |  |  |  |  |  |  |  |  |
| Women | 0.37 | 0.39 | 0.39 |  | 0.39 | 0.39 |  | 0.39 |
| Men | 0.41 | 0.42 | 0.43 |  | 0.42 | 0.42 |  | 0.43 |
| Total Sugars |  |  |  |  |  |  |  |  |
| Women | 0.43 | 0.45 | 0.45 |  | 0.44 | 0.45 |  | 0.45 |
| Men | 0.48 | 0.49 | 0.49 |  | 0.47 | 0.48 |  | 0.50 |
| Starch |  |  |  |  |  |  |  |  |
| Women | 0.33 | 0.34 | 0.34 |  | 0.35 | 0.34 |  | 0.34 |
| Men | 0.33 | 0.34 | 0.35 |  | 0.35 | 0.35 |  | 0.35 |
| Fibre |  |  |  |  |  |  |  |  |
| Women | 0.43 | 0.45 | 0.44 |  | 0.45 | 0.46 |  | 0.45 |
| Men | 0.45 | 0.46 | 0.47 |  | 0.47 | 0.47 |  | 0.46 |
| Alcohol |  |  |  |  |  |  |  |  |
| Women | 0.44 | 0.42 | 0.50 |  | 0.49 | 0.42 |  | 0.44 |
| Men | 0.43 | 0.42 | 0.51 |  | 0.51 | 0.42 |  | 0.43 |

^a^ Winter= phase 2 (Feb, Mar, Apr); Spring=phase 5 (Apr, May, Jun); Summer=phase 3 (Jul, Aug); Autumn=phase 4 (Oct, Nov, Dec).

**Supplementary Table S4.** Reproducibility of 24-hour dietary assessments by age group and Townsend Index of Deprivation. Intra-class correlations unadjusted for energy intake are presented

| Nutrient (g) | Age at First 24-Hr Assessment (years) | | | | | |  | Townsend Index (Quintiles) | | | | |
| --- | --- | --- | --- | --- | --- | --- | --- | --- | --- | --- | --- | --- |
|  | <45 | 45-49 | 50-54 | 55-59 | 60-64 | ≥65 |  | Least Deprived | 2 | 3 | 4 | Most Deprived |
| N |  |  |  |  |  |  |  |  |  |  |  |  |
| Women | 5,098 | 9,701 | 11,800 | 13,872 | 17,366 | 12,851 |  | 14,941 | 14,805 | 14,681 | 14,643 | 11,534 |
| Men | 3,783 | 6,488 | 7,624 | 9,794 | 14,582 | 13,850 |  | 12,786 | 12,084 | 11,312 | 11,028 | 8,846 |
| Energy (kJ) |  |  |  |  |  |  |  |  |  |  |  |  |
| Women | 0.32 | 0.32 | 0.32 | 0.34 | 0.34 | 0.37 |  | 0.33 | 0.33 | 0.33 | 0.34 | 0.35 |
| Men | 0.35 | 0.34 | 0.35 | 0.37 | 0.36 | 0.38 |  | 0.36 | 0.36 | 0.35 | 0.36 | 0.39 |
| Protein |  |  |  |  |  |  |  |  |  |  |  |  |
| Women | 0.29 | 0.28 | 0.29 | 0.29 | 0.29 | 0.30 |  | 0.29 | 0.27 | 0.28 | 0.30 | 0.32 |
| Men | 0.30 | 0.31 | 0.30 | 0.31 | 0.29 | 0.30 |  | 0.28 | 0.30 | 0.28 | 0.30 | 0.34 |
| Total Fat |  |  |  |  |  |  |  |  |  |  |  |  |
| Women | 0.29 | 0.29 | 0.30 | 0.31 | 0.32 | 0.34 |  | 0.30 | 0.31 | 0.31 | 0.31 | 0.32 |
| Men | 0.34 | 0.32 | 0.33 | 0.35 | 0.35 | 0.36 |  | 0.35 | 0.35 | 0.34 | 0.34 | 0.37 |
| Saturated Fat |  |  |  |  |  |  |  |  |  |  |  |  |
| Women | 0.29 | 0.31 | 0.32 | 0.32 | 0.34 | 0.37 |  | 0.32 | 0.32 | 0.34 | 0.32 | 0.34 |
| Men | 0.33 | 0.34 | 0.36 | 0.38 | 0.38 | 0.40 |  | 0.37 | 0.37 | 0.37 | 0.37 | 0.40 |
| Polyunsaturated Fat |  |  |  |  |  |  |  |  |  |  |  |  |
| Women | 0.20 | 0.21 | 0.20 | 0.22 | 0.22 | 0.24 |  | 0.21 | 0.22 | 0.21 | 0.22 | 0.23 |
| Men | 0.25 | 0.22 | 0.23 | 0.24 | 0.24 | 0.24 |  | 0.25 | 0.23 | 0.22 | 0.24 | 0.27 |
| Monounsaturated Fat |  |  |  |  |  |  |  |  |  |  |  |  |
| Women | 0.29 | 0.28 | 0.28 | 0.30 | 0.30 | 0.32 |  | 0.29 | 0.30 | 0.30 | 0.30 | 0.30 |
| Men | 0.33 | 0.30 | 0.32 | 0.33 | 0.32 | 0.35 |  | 0.33 | 0.33 | 0.32 | 0.32 | 0.35 |
| Carbohydrates |  |  |  |  |  |  |  |  |  |  |  |  |
| Women | 0.36 | 0.36 | 0.37 | 0.38 | 0.39 | 0.41 |  | 0.37 | 0.38 | 0.38 | 0.39 | 0.39 |
| Men | 0.39 | 0.40 | 0.40 | 0.43 | 0.41 | 0.43 |  | 0.41 | 0.41 | 0.40 | 0.42 | 0.43 |
| Total Sugars |  |  |  |  |  |  |  |  |  |  |  |  |
| Women | 0.42 | 0.40 | 0.42 | 0.43 | 0.45 | 0.47 |  | 0.43 | 0.44 | 0.43 | 0.44 | 0.45 |
| Men | 0.46 | 0.45 | 0.47 | 0.49 | 0.48 | 0.50 |  | 0.49 | 0.47 | 0.46 | 0.48 | 0.50 |
| Starch |  |  |  |  |  |  |  |  |  |  |  |  |
| Women | 0.31 | 0.32 | 0.32 | 0.33 | 0.33 | 0.34 |  | 0.32 | 0.33 | 0.31 | 0.34 | 0.36 |
| Men | 0.33 | 0.32 | 0.33 | 0.34 | 0.34 | 0.35 |  | 0.33 | 0.33 | 0.33 | 0.33 | 0.36 |
| Fibre |  |  |  |  |  |  |  |  |  |  |  |  |
| Women | 0.42 | 0.41 | 0.43 | 0.44 | 0.44 | 0.44 |  | 0.42 | 0.43 | 0.43 | 0.45 | 0.45 |
| Men | 0.45 | 0.43 | 0.45 | 0.45 | 0.46 | 0.45 |  | 0.45 | 0.44 | 0.44 | 0.46 | 0.48 |
| Alcohol |  |  |  |  |  |  |  |  |  |  |  |  |
| Women | 0.35 | 0.39 | 0.42 | 0.44 | 0.49 | 0.52 |  | 0.43 | 0.42 | 0.45 | 0.46 | 0.46 |
| Men | 0.38 | 0.38 | 0.42 | 0.47 | 0.48 | 0.52 |  | 0.44 | 0.45 | 0.45 | 0.47 | 0.49 |

**Supplementary Table S5.** Reproducibility of 24-hour dietary assessment by BMI at recruitment. Intra-class correlations unadjusted for energy intake are presented

| Nutrient (g) | BMI at Recruitment (m/kg^2^) | | | | |
| --- | --- | --- | --- | --- | --- |
|  | Underweight | Normal | Overweight | Obese I | Obese II/III |
| N |  |  |  |  |  |
| Women | 610 | 32,588 | 24,206 | 8,919 | 4,263 |
| Men | 119 | 16,762 | 27,443 | 9,259 | 2,417 |
| Energy (kJ) |  |  |  |  |  |
| Women | 0.43 | 0.35 | 0.32 | 0.32 | 0.33 |
| Men | 0.46 | 0.38 | 0.36 | 0.35 | 0.35 |
| Protein |  |  |  |  |  |
| Women | 0.39 | 0.31 | 0.27 | 0.27 | 0.27 |
| Men | 0.37 | 0.32 | 0.29 | 0.28 | 0.32 |
| Total Fat |  |  |  |  |  |
| Women | 0.41 | 0.32 | 0.30 | 0.30 | 0.30 |
| Men | 0.47 | 0.36 | 0.34 | 0.33 | 0.34 |
| Saturated Fat |  |  |  |  |  |
| Women | 0.46 | 0.35 | 0.31 | 0.31 | 0.30 |
| Men | 0.53 | 0.39 | 0.37 | 0.36 | 0.38 |
| Polyunsaturated Fat |  |  |  |  |  |
| Women | 0.31 | 0.23 | 0.20 | 0.20 | 0.22 |
| Men | 0.29 | 0.27 | 0.23 | 0.23 | 0.19 |
| Monounsaturated Fat |  |  |  |  |  |
| Women | 0.37 | 0.31 | 0.29 | 0.29 | 0.28 |
| Men | 0.45 | 0.34 | 0.33 | 0.31 | 0.32 |
| Carbohydrates |  |  |  |  |  |
| Women | 0.47 | 0.40 | 0.37 | 0.37 | 0.35 |
| Men | 0.49 | 0.43 | 0.41 | 0.40 | 0.39 |
| Total Sugars |  |  |  |  |  |
| Women | 0.53 | 0.45 | 0.43 | 0.42 | 0.41 |
| Men | 0.48 | 0.50 | 0.48 | 0.45 | 0.46 |
| Starch |  |  |  |  |  |
| Women | 0.50 | 0.35 | 0.32 | 0.30 | 0.31 |
| Men | 0.58 | 0.37 | 0.33 | 0.32 | 0.30 |
| Fibre |  |  |  |  |  |
| Women | 0.53 | 0.45 | 0.42 | 0.41 | 0.42 |
| Men | 0.63 | 0.49 | 0.44 | 0.41 | 0.43 |
| Alcohol |  |  |  |  |  |
| Women | 0.49 | 0.44 | 0.44 | 0.44 | 0.44 |
| Men | 0.66 | 0.49 | 0.45 | 0.43 | 0.43 |

**Supplementary Table S6.** Mean intake (g [SD]) of food group intake and average score of dietary patterns on 24-hour dietary assessments (24-Hr) and short FFQ in UK Biobank

|  | Men | |  | Women | |
| --- | --- | --- | --- | --- | --- |
|  | 24-Hr | Short FFQ |  | 24-Hr | Short FFQ |
| **Food Groups** |  |  |  |  |  |
| Total Meat | 101.9 (76.2) | 92.9 (55.1) |  | 83.6 (66.2) | 76.7 (47.6) |
|  |  |  |  |  |  |
| Cheese | 17.5 (21.7) | 15.8 (15.5) |  | 16.8 (20.1) | 14.6 (15.4) |
|  |  |  |  |  |  |
| Fish | 27.0 (46.5) | 35.3 (31.8) |  | 26.5 (42.0) | 36.3 (30.8) |
|  |  |  |  |  |  |
| Bread & Cereal | 162.8 (96.5) | 127.4 (73.2) |  | 130.7 (82.8) | 115.2 (72.1) |
|  |  |  |  |  |  |
| Fruit | 191.4 (161.2) | 209.5 (164.6) |  | 218.1 (164.1) | 247.3 (162.3) |
|  |  |  |  |  |  |
| Vegetables | 185.5 (159.2) | 168.2 (121.9) |  | 229.8 (176.0) | 180.5 (112.1) |
| **Dietary Patterns** |  |  |  |  |  |
| Mediterranean ^a^ | 3.0 (1.2) |  |  | 3.1 (1.2) |  |
|  |  |  |  |  |  |
| Vegetarian (N [%])^b^ | 3217 (3.4) | 3055 (1.3) |  | 6241 (5.4) | 6081 (2.2) |

^a^ (range=0-9, mean ranking [SD]) Greater than median intake (servings/d) for vegetables, legumes, fruit, nuts, fish, wholegrains, ratio of monounsaturated to saturated fat; less than median intake for red and processed meat; and between 5-25 g/d for ethanol. ^b^ Intake for vegetarian dietary pattern is N (%) at any point in 24-hr dietary assessment or at baseline in the short FFQ.
